# Mitochondrial Defects in Fibroblasts of Pathogenic *MAPT* Patients

**DOI:** 10.3389/fcell.2021.765408

**Published:** 2021-11-03

**Authors:** Vinita Bharat, Chung-Han Hsieh, Xinnan Wang

**Affiliations:** Department of Neurosurgery, Stanford University School of Medicine, Stanford, CA, United States

**Keywords:** ER, mitochondria, Miro, parkinsonism, FTLD, mitophagy, tau, *MAPT*

## Abstract

Mutations in *MAPT* gene cause multiple neurological disorders, including frontal temporal lobar degeneration and parkinsonism. Increasing evidence indicates impaired mitochondrial homeostasis and mitophagy in patients and disease models of pathogenic *MAPT*. Here, using *MAPT* patients’ fibroblasts as a model, we report that disease-causing *MAPT* mutations compromise early events of mitophagy. By employing biochemical and mitochondrial assays we discover that upon mitochondrial depolarization, the recruitment of LRRK2 and Parkin to mitochondria and degradation of the outer mitochondrial membrane protein Miro1 are disrupted. Using high resolution electron microscopy, we reveal that the contact of mitochondrial membranes with ER and cytoskeleton tracks is dissociated following mitochondrial damage. This membrane dissociation is blocked by a pathogenic *MAPT* mutation. Furthermore, we provide evidence showing that tau protein, which is encoded by *MAPT* gene, interacts with Miro1 protein, and this interaction is abolished by pathogenic *MAPT* mutations. Lastly, treating fibroblasts of a *MAPT* patient with a small molecule promotes Miro1 degradation following depolarization. Altogether, our results show molecular defects in a peripheral tissue of patients and suggest that targeting mitochondrial quality control may have a broad application for future therapeutic intervention.

## Introduction

Mitochondria are a vital organelle to support neuronal function and survival. Emerging evidence has revealed mitochondrial malfunction in a broad spectrum of neurological disorders (Misgeld and Schwarz, 2017). One such neurological condition is tauopathy, which is shared by multiple neurodegenerative diseases such as Alzheimer’s disease (AD), progressive supranuclear palsy (PSP), frontal temporal lobar degeneration (FTLD), and parkinsonism (Spillantini and Goedert, 2013; Medina, 2018). Tauopathy is featured with intracellular neurofibrillary tangles. The main constituent of those filaments is tau protein, which is encoded by *MAPT* gene. Mutations in *MAPT* gene result in production of abnormal tau protein and promote tangle formation. Therefore, pathogenic *MAPT* mutations are detrimental to neuronal integrity and function (Fuster-Matanzo et al., 2018). In several mouse models, overexpression of tau affects mitochondrial distribution, transport, and clearance (Thies and Mandelkow, 2007; Zempel and Mandelkow, 2015; Hu et al., 2016). Recent reports have shown that mutant tau impairs mitophagy (Hu et al., 2016; Cummins et al., 2019; Fang et al., 2019), a mitochondria-specific autophagy process, likely by inhibiting Parkin recruitment to damaged mitochondria (Hu et al., 2016; Cummins et al., 2019). Further characterization of mitophagy steps affected by pathogenic *MAPT* would help us understand better the underlying mechanisms and potential implications for disease pathogenesis.

**FIGURE 1:**
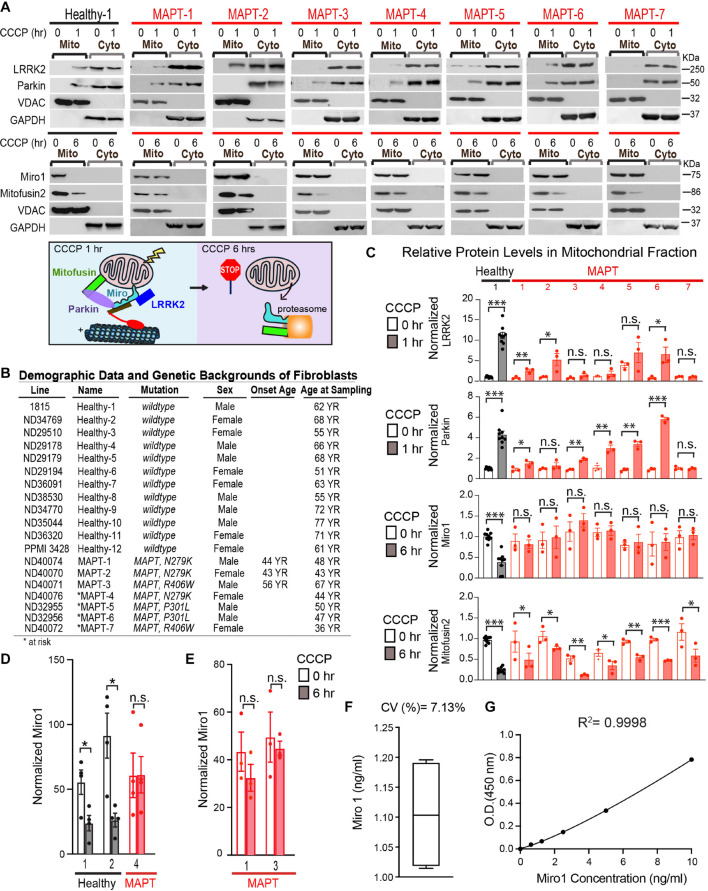
Mitochondrial protein changes following CCCP treatment in fibroblasts. **(A)** Mitochondrial (Mito) and cytosolic (Cyto) fractions were immunoblotted as indicated. Below: Schematic representation of our readouts. **(B)** Demographic and genetic information of all cell lines used in this study or described in Text. **(C)** Quantifications of mitochondrial protein levels. The intensity of each band in the mitochondrial fraction is normalized to that of the mitochondrial loading control VDAC from the same blot and expressed as a fraction of Mean of Healthy-1 with DMSO treatment; this control was included in every experiment. Student *T* Test is performed for comparing normalized band intensities within the same subject (DMSO vs. CCCP). *N* = 3–9 independent experiments. Please note that Healthy-2 to 12 show similar mitochondrial protein responses to CCCP as Healthy-1, previously published in Hsieh et al. (2019). **(D,E)** ELISA of Miro1 protein. Comparison within the same subject. Mann-Whitney *U* Test. **(D)** *N* = 4 with duplicates each time. **(E)** *N* = 3. **(F)** Intra-plate variability of ELISA shown in **(E)**, measured by running the same fibroblast sample 4 times in the same plate. **(G)** The standard curve for **(E)** is shown. Sigmoidal 4PL is used. ^∗^*P* < 0.05, ^∗∗^*P* < 0.01, ^∗∗∗^*P* < 0.001. n.s.: not significant.

Upon mitochondrial damage, outer mitochondrial membrane (OMM) proteins rapidly undergo proteasome degradation, prior to lysosomal digestion of the remaining mitochondrion (Chan et al., 2011). This initial step is thought to facilitate the dissociation of a damaged mitochondrion from the rest of the healthy mitochondrial network to halt the spread of damage. Particularly, the removal of Mitofusin protein has been shown to promote mitochondrial fragmentation and the separation of mitochondria-ER tethering (Poole et al., 2010; Tanaka et al., 2010; Hsieh et al., 2016; McLelland et al., 2018); this step can sever a damaged part from a large, otherwise healthy mitochondrion and block ER contact with mitochondrial damage. In addition, the removal of Miro protein uncouples mitochondria from motors and microtubule tracks to prevent mitochondria from moving around (Wang et al., 2011). Importantly, we have recently found that removing Miro is also essential for static mitochondria to undergo mitophagy (Hsieh et al., 2016, 2019; Shaltouki et al., 2018), pinpointing a role for Miro in additional steps of mitophagy. The degradation of these OMM proteins following mitochondrial depolarization is mediated by at least two pathways – LRRK2 and the PINK1-Parkin axis (Wang et al., 2011; Chen and Dorn, 2013; Hsieh et al., 2016; Figure 1A). Mutations in *LRRK2*, *PINK1*, or *Parkin* cause familial Parkinson’s disease (PD) (Kitada et al., 1998; Bonifati, 2002; Valente et al., 2004; Zimprich et al., 2004). However, whether the removal of these OMM proteins from damaged mitochondria is affected by pathogenic *MAPT* mutations remains largely elusive.

Skin fibroblast cells cultured directly from patients have emerged as an outstanding model to study disease-relevant molecular defects, because these cells retain the identical genetic backgrounds of patients and contain molecular and mitochondrial pathologies (Smith et al., 2016; Di Nottia et al., 2017; Fitzgerald et al., 2017; Garcia-Sanz et al., 2017; Teves et al., 2017; Verma et al., 2017; Juarez-Flores et al., 2018; Bonello et al., 2019; Korecka et al., 2019). In addition, skin fibroblasts can be obtained from patients by a minimally invasive, painless procedure (Le et al., 2017), and thus are a convenient source for developing biomarkers and diagnostic assays. A biomarker in biopsied skin tissues may originate from an alternative biological process to that in body fluids, providing an additional and possibly more reliable (Le et al., 2017) predicative value. In this study, we examined several mitochondrial behaviors in fibroblasts of *MAPT* patients, and found clear evidence of impairments in these molecular and cellular processes.

## Materials and Methods

### Cell Culture and Biochemistry

Fibroblasts were obtained under an MTA from the National Institute of Neurological Disorders and Stroke (NINDS) human and cell repository or the Parkinson’s Progression Markers Initiative (PPMI), which is in a partnership with multiple institutions that approved study protocols, ensured consent from donors, and deposited fibroblasts. All available lines were acquired from NINDS at the time of purchase. Fibroblast and HEK cell culture, immunoprecipitation (IP), and mitochondrial purification were described in Hsieh et al. (2016). Briefly, CCCP in DMSO or the same volume of DMSO treated fibroblasts were lifted by a cell scraper, and mechanically homogenized with a Dounce homogenizer in 750 μl isolation buffer (200 mM sucrose, 10 mM TRIS/MOPS, pH 7.4). After centrifugation at 500 *g* for 10 min, crude supernatant was spun at 10,000 g for 10 min to pellet intact mitochondria. Mitochondrial pellet was washed twice with isolation buffer. After this step, supernatant was named “cytosolic fraction (Cyto),” and pellet was resuspended in 50 μl lysis buffer (50 mM Tris pH 8.0, 150 mM NaCl, and 1% Triton X-100–T8787, Sigma-Aldrich) with 0.25 mM phenylmethanesulfonyl fluoride (P7626, Sigma-Aldrich) and protease inhibitors (Roche) named “mitochondrial fraction (Mito).” Samples were mixed 1:1 with 2 × laemmli buffer (4% SDS, 20% Glycerol, 120 mM Tris–HCl, 0.02% bromophenol blue, 700 mM 2-mercaptoethanol) and boiled for 5 min prior to being loaded (Mito:Cyto = 25:1) into an SDS-PAGE. pRK5-EGFP-tau (#46904, addgene), pRK5-EGFP-tau-P301L, pRK5-EGFP-tau-N279K, and pRK5-EGFP-tau-R406W (the latter three were custom-made by Synbio Technology) were used for transfection by the calcium phosphate transfection protocol (Wang and Schwarz, 2009). Transferred membranes were first blocked overnight in phosphate-buffered saline (PBS) containing 5% fat-free milk and 0.1% tween-20 at 4°C, and then incubated with the following primary antibodies: mouse anti-Miro1 (WH0055288M1, Sigma-Aldrich) at 1:1,000, rabbit anti-Miro1 (HPA010687, Sigma-Aldrich) at 1:1,000, rabbit anti-VDAC (4661S, Cell Signaling Technology) at 1:1,000, mouse anti-Mitofusin2 (H00009927-M01, Abnova) at 1:1,000, mouse anti-Parkin (sc32282, Santa Cruz Biotechnology) at 1:500, rabbit anti-LRRK2 (NB300-268, Novus Biologicals) at 1:500, rabbit anti-GAPDH (5174S, Cell Signaling Technology) at 1:3,000, rabbit anti-β-actin (4967S, Cell Signaling Technology) at 1:1,000, rabbit anti-GFP (A11122, Invitrogen) at 1:750, mouse anti-ATP5β (ab14730, Abcam) at 1:100, rabbit anti-Calreticulin (2891, Cell Signaling Technology) at 1:1,000, mouse anti-Golgi (NB600-412, Novus Biologicals) at 1:100, or mouse anti-Myc (9E10/sc-40, Santa Cruz Biotechnology) at 1:100, at 4°C overnight in blocking buffer. HRP-conjugated goat anti-mouse or rabbit IgG (Jackson ImmunoResearch Laboratories) were used at 1:5,000–10,000. West Dura ECL Reagents (34075, GE Healthcare) were used for ECL immunoblotting. Membranes were exposed to UltraCruz autoradiography films (Santa Cruz Biotechnology) and developed on a Konica Minolta SRX-101A developer or scanned using a Bio-Rad ChemiDoc XRS system.

All experiments were performed in a blinded format, and the identities of the lines were un-blinded after the experiments. For Figure 1C, the intensities of protein bands were measured by ImageJ (ver. 1.48V, NIH). The intensity of each band in the mitochondrial fraction was normalized to that of the mitochondrial loading control VDAC from the same blot, and expressed as a fraction of Mean of Healthy-1 with DMSO treatment (Hsieh et al., 2016); this control was included in every independent experiment. The band intensities of VDAC were not significantly different among all fibroblast lines and conditions (*P* > 0.8, One-Way ANOVA Post Hoc Tukey Test).

### Enzyme-Linked Immunosorbent Assay (ELISA)

All experiments were performed as blinded tests. A 40 μM CCCP in DMSO or the same volume of DMSO alone was applied to fibroblasts for 6 h, and then cells were lysed in lysis buffer (100 mM Tris, 150 mM NaCl, 1 mM EGTA, 1 mM EDTA, 1% Triton X-100, 0.5% Sodium deoxycholate) with protease inhibitor cocktail (539134, Calbiochem). Cell debris was removed by centrifugation at 17,000 *g* for 10 min at 4°C. Details of ELISA are in Supplementary Methods.

### Transmission Electron Microscopy (TEM)

Cells were grown on ∼10 mm Aclar discs in 37°C incubator with 5% CO_2_ till 95–100% confluent. Each Aclar disc was then transferred separately into an Eppendorf tube with 1 ml of fixative solution (2% glutaraldehyde and 4% paraformaldehyde in 0.1 M sodium cacodylate buffer) and incubated for an hour. These cells were kept on ice until processed for imaging. Fixative was removed and 1% OsO_4_ in ddH_2_O was added. Samples were then gently shaken and incubated for 1 h at 4°C. Incubated samples were washed three times with cold ddH_2_O for 5 min each. Samples were then stained with 1% uranyl acetate for 2 h at 4°C. Stained samples were dehydrated, first with 50% EtOH for 10 min, next 70% EtOH for 10 min, and lastly 95% EtOH for 10 min. Samples were allowed to warm up to room temperature before two more dehydration procedures with 100% EtOH for 10 min each. The final dehydration was performed with acetonitrile for 15 min. The dehydration mixture was then replaced with Embed 812 medium (44% Embed 812, 35% DDSA, 18% NMA, 3% BDMA) and acetonitrile (1:1 ratio) for 1 h. The ratio was then increased to 2:1 (Embed 812 medium to acetonitrile) for overnight incubation. Next, the mixture was replaced with 100% Embed 812 medium and incubated for 2 h before samples were placed in molds for overnight settling. Settled samples were polymerized in a 65°C oven for 24 h before being sectioned for imaging. Imaging was performed using a Jeol TEM 1400 microscope, and images were acquired at 5,000×. Images were saved as.dc3 files. Analysis of.dc3 images was performed using ImageJ.

### Statistics

Throughout the paper, the distribution of data points was expressed as box-whisker, or dot-plot with Mean ± SEM, except otherwise stated. Box center line is median and box limits are upper and lower quartiles. One-Way or Two-Way ANOVA Post Hoc Tukey Test was performed for comparing multiple groups. Mann-Whitney *U* or *T* Test was performed for comparing two groups. Chi-Square Test was performed for Figure 3B because the data was categorical. Statistical analyses were performed using the Prism software (ver. 8.01, GraphPad) or Excel (ver. 16.51). The number of experimental replications (n) can be found in Figure Legends. No statistical methods were used to predetermine sample sizes, but the number of experiments and biological replicates was chosen based on the nature of the experiments (it is usually difficult to assess an outcome that follows a normal distribution in our experiments), degree of variations, and published papers describing similar experiments. We did not exclude any data. ^∗^*P* < 0.05, ^∗∗^*P* < 0.01, ^∗∗∗^*P* < 0.001, for all Figures.

## Results

### The LRRK2 and PINK1-Parkin Pathways Are Affected in Selective Fibroblast Lines of Pathogenic *MAPT* Patients

We have identified two parallel molecular pathways essential for removing Miro from the OMM of depolarized mitochondria–LRRK2 and the PINK1-Parkin axis (Wang et al., 2011; Hsieh et al., 2016, 2019). In addition, the OMM protein Mitofusin2 is a target of the PINK1-Parkin pathway, but not of LRRK2, for depolarization-triggered degradation (Poole et al., 2010; Tanaka et al., 2010; Hsieh et al., 2016). We have previously found that in 12 healthy control fibroblast lines, CCCP treatment, which depolarizes the mitochondrial membrane potential (ΔΨm), for only 1 h triggers the recruitment of a small fraction of cytosolic LRRK2 and Parkin to mitochondria, prior to Miro1 and Mitofusin2 removal at 6 h detected by Western blotting (Figures 1A–C; Supplementary Figure 1A; Hsieh et al., 2016, 2019). Antibodies against LRRK2, Parkin, and Miro1 have been validated in human cells lacking the corresponding genes (Hsieh et al., 2016). We then examined whether LRRK2 and Parkin recruitment to damaged mitochondria was impaired in 7 fibroblast lines of FTLD and parkinsonism patients or at-risk individuals with *MAPT* mutations, obtained from the NINDS human and cell repository (Figure 1B; Supplementary Table 1). At-risk subjects are younger asymptomatic family members of probands and carry the same genetic mutations. In contrast to healthy controls, 4 *MAPT* lines failed to significantly recruit LRRK2 to mitochondria, and 2 lines failed to recruit Parkin (Figures 1A–C). One line (MAPT-7, R406W) failed to recruit both proteins (Figures 1A–C). Basal levels of LRRK2 and Parkin were comparable among all lines (*P* > 0.8). Collectively, our results provide evidence indicating that the LRRK2 and PINK1-Parkin pathways are affected in selective *MAPT* patients’ fibroblast lines.

### Miro1 Is Resistant to Removal From Depolarized Mitochondria in Fibroblasts of *MAPT* Patients

Because the failure to relocate LRRK2 or Parkin to damaged mitochondria disrupts the following removal of Miro1 or Mitofusin2 (Hsieh et al., 2016), we next examined Miro1 and Mitofusin2 protein levels in the mitochondrial fractions of these cells upon depolarization. In healthy controls at 6 h following CCCP treatment, both Miro1 and Mitofusin2 are removed from damaged mitochondria detected by Western blotting (Figures 1A–C); this time point is earlier than the completion of mitophagy when multiple mitochondrial markers are degraded (Figure 1A; Hsieh et al., 2016, 2019). Notably, we discovered a unifying impairment in removing Miro1 from the mitochondrial fractions at 6 h after CCCP treatment in all 7 *MAPT* lines (Figures 1A–C). By contrast, Mitofusin2 was significantly degraded in the mitochondrial fractions after CCCP treatment in all *MAPT* lines, just as in control cells (Figures 1A–C). Basal protein levels of Miro1 and Mitofusin2 were largely comparable among all lines (Figure 1C; *P* > 0.09). For all experiments, the cell passaging numbers were within the range of 5–19 which had no influence on the phenotypes (Hsieh et al., 2019). We next validated the result from Western blotting by an alternative assay: ELISA (Hsieh et al., 2019; Nguyen et al., 2021). We tested 3 *MAPT* lines and consistently observed the resistance of Miro1 to degradation upon CCCP treatment (Figures 1D–G; Hsieh et al., 2019). Taken together, our results show that the failure to remove Miro1 from damaged mitochondria is a common molecular defect associated with pathogenic *MAPT* mutations.

### The Dissociation of Mitochondria From ER and Cytoskeleton Tracks Is Impaired in Pathogenic *MAPT* Fibroblasts

**FIGURE 2:**
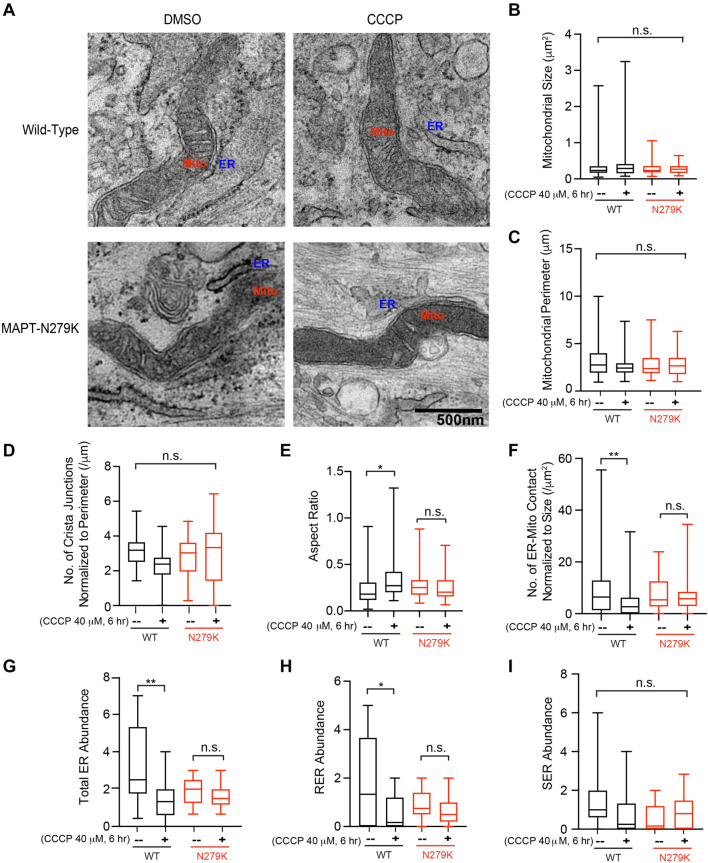
Ultrastructural changes of mitochondria in fibroblasts. **(A)** Representative TEM images of Healthy-6 (WT) and MAPT-1 (MAPT-N279K) with and without CCCP treatment. Scale bar: 500 nm. **(B–I)** Quantifications from images as in **(A)**. **(B)** Quantification of mitochondrial size (minor × major diameter). **(C)** Quantification of mitochondrial perimeter. **(D)** Quantification of cristae junction number normalized to mitochondrial perimeter. **(E)** Quantification of aspect ratio (minor/major diameter). **(F)** Quantification of ER-mitochondrial contact number normalized to mitochondrial size (contact is defined where the distance between ER and mitochondrial membranes is less than 10 nm). **(G)** Quantification of total ER abundance (the total number of ER-mitochondrial contact divided by the total number of mitochondria per image). **(H)** Quantification of rough ER (RER) abundance. Similar to **(G)** but only RER is counted. **(I)** Quantification of smooth ER (SER) abundance. Similar to **(G)** but only SER is counted. *N* = 57 (WT, DMSO), 51 (WT, CCCP), 53 (MAPT-N279K, DMSO), and 64 (MAPT-N279K, CCCP) mitochondria from 15 different images from 3 independent cultures. Two-Way ANOVA Post Hoc Tukey Test. *P < 0.05, **P < 0.01. n.s.: not significant.

**FIGURE 3:**
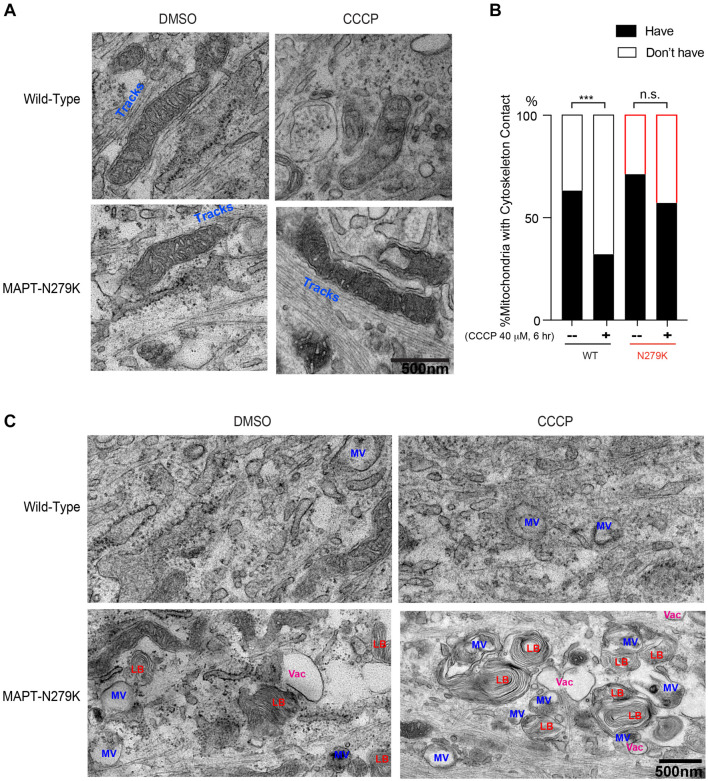
Phenotypes of cytoskeleton and other organelles in fibroblasts. **(A)** Representative TEM images of Healthy-6 (WT) and MAPT-1 (MAPT-N279K) with and without CCCP treatment, showing cytoskeleton tracks (Tracks) next to mitochondria. **(B)** From images as in **(A)**, the percentage of total mitochondria with or without adjacent cytoskeleton tracks is counted. *N* = 30 (WT, DMSO), 28 (WT, CCCP), 31 (MAPT-N279K, DMSO), and 36 (MAPT-N279K, CCCP) mitochondria from 15 images from 3 independent cultures. Chi Square Test. **(C)** Representative TEM images of Healthy-6 (WT) and MAPT-1 (MAPT-N279K) with and without CCCP treatment, showing vacuole-like structures (Vac), structures similar to multi-vesicular bodies (MV), and structures similar to lamellar bodies (LB). Scale bars: 500 nm. ***P < 0.001. n.s.: not significant.

**FIGURE 4:**
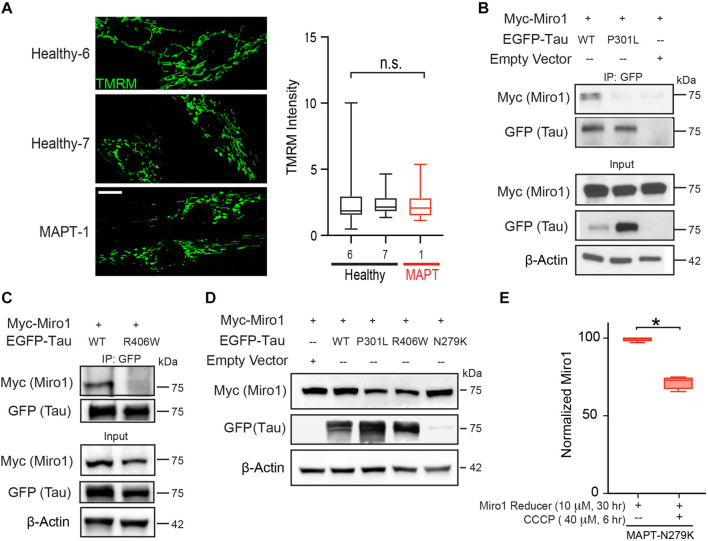
The mitochondrial network in fibroblasts, tau interaction with Miro1, and effect of Miro1 Reducer. **(A)** Confocal images show mitochondria stained with TMRM in fibroblasts. The mean TMRM intensity of each cell is normalized to the background intensity and quantified. *N* = 43 (Healthy-6), 46 (Healthy-7), and 42 (MAPT-1) cells from 4 to 5 images per coverslip from 3 coverslips. One-Way ANOVA Post Hoc Tukey Test. Scale bar: 10 μM. **(B,C)** Co-IP with anti-GFP from HEK cells transfected as indicated. **(D)** HEK cells transfected with different tau and Miro1 constructs were lysed and blotted. **(E)** ELISA of Miro1 protein in MAPT-1 fibroblasts treated as indicated, *N* = 4. Mann-Whitney *U* Test. *P < 0.05. n.s.: not significant.

In order to examine mitochondrial changes at the ultrastructural level, we performed TEM on a healthy and a *MAPT* fibroblast line used in Figure 1, with or without CCCP treatment for 6 h (Figure 2A). We found that the mitochondrial size, perimeter, and crista junction number were indistinguishable among all conditions (Figures 2B–D), whereas the aspect ratio (minor to major diameter) was increased in healthy control with CCCP treatment (Figure 2E), indicating mitochondrial rounding in this condition. Strikingly, we found that following CCCP treatment the ER-mitochondrial contact number, particularly for rough ER (RER), was significantly reduced in healthy fibroblasts; however, it was not altered at the same time point in *MAPT* fibroblasts (Figures 2A,F–I). This result suggests that mitochondrial depolarization causes the separation of mitochondria and ER contact, consistent with a previous report (McLelland et al., 2018). This study also implicates that the mitochondrial and ER separation is essential for the following mitophagy, and blocking their dissociation, as seen in *MAPT* fibroblasts (Figure 2), could lead to mitophagy impairment (McLelland et al., 2018), observed in other tau models as well (Hu et al., 2016; Cummins et al., 2019; Fang et al., 2019). In addition, we saw mitochondria frequently juxtaposed with cytoskeleton (actin or microtubule) tracks in healthy control fibroblasts and this association was significantly reduced following CCCP treatment (Figures 3A,B), showing that damaged mitochondria are sequestered from cytoskeleton networks. However, *MAPT* fibroblasts failed to exhibit this response (Figures 3A,B). Furthermore, *MAPT* fibroblasts displayed the widespread presence of structures resembling lamellar bodies (LB) and multivesicular bodies (MV) under both basal and depolarized conditions (Figure 3C), indicating possible imbalance in proteostasis and lipid homeostasis. The global mitochondrial network or ΔΨm visualized by TMRM staining was indistinguishable among healthy and *MAPT* fibroblasts at baseline (Figure 4A). Together, our results show that the dissociation of damaged mitochondria from ER and cytoskeleton networks is blocked in fibroblasts of a *MAPT* patient.

### *MAPT* Mutations Disrupt Tau Interaction With Miro1

One hypothesis that could explain our TEM observations is that normal *MAPT* is essential to facilitate the separation of mitochondria from ER and cytoskeleton at the early stage of mitophagy. Interestingly, Miro can localize to the ER-mitochondrial contact sites (Kornmann et al., 2011; Stroud et al., 2011) and anchors mitochondria to microtubule and actin tracks (Stowers et al., 2002; Glater et al., 2006; Wang and Schwarz, 2009; Morlino et al., 2014; Lopez-Domenech et al., 2018; Oeding et al., 2018). Notably, Miro is quickly removed from depolarized mitochondria (Chan et al., 2011; Wang et al., 2011; Birsa et al., 2014), and our earlier results showed a unifying impairment in removing Miro1 from damaged mitochondria in all 7 *MAPT* lines (Figure 1), suggesting that Miro1 and tau may coordinate to ensure an efficient mitophagy process. We next determined whether tau could physically interact with Miro1. By performing co-immunoprecipitation (co-IP) from HEK cells transfected with tau and Miro1 constructs, we found that wild-type tau, but not tau-P301L or tau-R406W, interacted with Miro1 (Figures 4B,C; Supplementary Figure 1B). Tau-N279K protein was not well-expressed in HEK cells (Figure 4D) though the same amount of DNA was transfected as tau-P301L or tau-R406W, which did not allow reliable co-IP experiments. Therefore, mutant tau compromises its ability to bind to Miro1.

### Miro1 Reducer Rescues the Phenotype of Miro1 Retention in Pathogenic *MAPT* Fibroblasts

The failure to remove Miro1 from damaged mitochondria in fibroblasts of *MAPT* patients (Figure 1) is reminiscent of that observed in fibroblasts of PD patients (Hsieh et al., 2019). We have previously discovered a small molecule (named Miro1 Reducer or MR3) that binds to and destabilizes human Miro1 protein (Hsieh et al., 2019; Li et al., 2021). Treating fibroblasts of PD patients with Miro1 Reducer rescues the phenotype of Miro1 accumulation on damaged mitochondria, and applying Miro1 Reducer to human neuron and fly models of PD ameliorates Parkinson’s relevant phenotypes, without affecting Miro1’s overall GTPase activity or other mitochondrial proteins including Miro2, Mitofusin, OPA1, VDAC, and ATP5β (Hsieh et al., 2019). We examined whether Miro1 Reducer could also promote Miro1 degradation in *MAPT* fibroblasts. We administered Miro1 Reducer to MAPT-1 line which exhibited the phenotype of Miro1’s resistance to degradation upon CCCP treatment, shown earlier (Figure 1). We found that Miro1 Reducer treatment caused significant Miro1 degradation following CCCP treatment detected by ELISA (Figure 4E), just like the response of Miro1 in healthy control fibroblasts (Figure 1). These results suggest that pharmacologically targeting Miro1 could be tested as a therapeutic approach in tauopathy models.

## Discussion

In this study, we have revealed mitochondrial molecular and membrane dynamic defects upon depolarization in fibroblasts of pathogenic *MAPT* patients. The failure to recruit LRRK2 or Parkin to damaged mitochondria and to remove Miro1 from the OMM could slow or impair the following mitophagy leading to an accumulation of damaged mitochondria (Hsieh et al., 2016, 2019; Shaltouki et al., 2018). In addition, the failure to separate mitochondria from ER and cytoskeleton networks could also hinder the mitophagy process (Wang et al., 2011; McLelland et al., 2018). The discovery of these mitochondrial defects in a peripheral tissue of patients may aid in biomarker development, as well as shed light on pathogenic processes in tauopathy.

Our work points to a crucial role for tau in maintaining dynamics of ER-mitochondrial contact sites. Consistently, a previous study has shown that mutant tau stabilizes ER-mitochondrial tethering in mouse models (Perreault et al., 2009). Mechanistically, tau may conduct this role via Miro. Here, we have shown that wild-type tau physically interacts with Miro1 and mutant tau disrupts this interaction. Both tau and Miro can reside at the ER-mitochondrial contact sites (Perreault et al., 2009; Kornmann et al., 2011; Stroud et al., 2011; Cieri et al., 2018). Importantly, Miro is quickly detached from the OMM of depolarized mitochondria to facilitate the following mitophagy (Chan et al., 2011; Wang et al., 2011; Birsa et al., 2014). Because the classical role of Miro is to anchor mitochondria to microtubule motors to mediate mitochondrial transport, eliminating Miro stops damaged mitochondrial motility and limits the spread of damage. It is highly possible that in addition to halting mitochondrial transport, removing Miro from damaged mitochondria helps the dissociation of ER and mitochondrial tethering, which is similarly essential to ensure efficient mitophagy (McLelland et al., 2018). This hypothesis is in line with our previous observations that Miro degradation is required for all mitochondria, both motile and static, to undergo mitophagy (Hsieh et al., 2016, 2019; Shaltouki et al., 2018). Therefore, our studies suggest that Miro may have multiple roles in mitophagy: on one hand ablating Miro uncouples damaged mitochondria from microtubules, and on the other hand deleting Miro separates damaged mitochondria from ER, resulting in the quarantine of mitochondrial damage. Wild-type tau might help remove Miro from these mitochondrial contact sites and mutant tau might lose this function. Further work is warranted to reveal the underlying molecular mechanisms. For example, how exactly does tau facilitate Miro removal from the mitochondrial surface? Do other mitophagy players such as Mitofusin, Parkin, and LRRK2 play a part? Parkin and Mitofusin are also important for the integrity of ER-mitochondrial contact sites (Basso et al., 2018; McLelland et al., 2018), and LRRK2 interplays with tau (Bardai et al., 2018). Our results have shown that LRRK2 or Parkin relocation to damaged mitochondria is compromised in selective fibroblast lines of *MAPT* (Figure 1). However, this defect seems to be cell line-specific, rather than mutation-specific. For example, LRRK2 recruitment is normal in two *MAPT* lines with the N279K mutation, but impaired in a third *MAPT* line with the same mutation (Figure 1C). In addition, MAPT-1 or MAPT-6 cell line is able to recruit both LRRK2 and Parkin, but still fails to degrade Miro1 (Figure 1C). Perhaps normal tau is essential for the enzymatic activities of LRRK2 and Parkin, rather than their relocation to damaged mitochondria. Or LRRK2 and Parkin function in parallel to tau to mediate Miro removal.

Detecting endogenous LRRK2 in human fibroblasts has yielded variable results among different laboratories. It is possible that different antibodies, experimental procedures, and handlings may have contributed to the discrepancies. It is important to validate the specificity of an antibody using a knockout cell line. The interaction of LRRK2 with mitochondria needs further investigations. State-of-art techniques such as super-resolution microscopy, immuno-gold TEM, and mitochondrial sub-fractionation et cetera can help us understand the nature of their interaction. Although we have observed robust phenotypes with TEM in one patient’s cell line, future work is required to extend the TEM study to additional *MAPT* patients’ lines. Fibroblasts provide a great value for biomarker studies, yet employing neuronal models is essential to understand disease mechanisms. Given the wide involvement of these mitophagy players including LRRK2, Parkin, tau, and Miro1 in age-dependent neurodegenerative diseases, it would be imperative to examine whether the failure to disconnect damaged mitochondria from ER and cytoskeleton tracks underlies additional diseases including AD and PSP. Testing Miro1 reducers in human neuron and *in vivo* models of tauopathy would be crucial to determine the therapeutic potential of Miro1. Together, our results support a convergent role of mitochondrial quality control in age-dependent neurodegeneration (Menzies et al., 2015; Pickrell and Youle, 2015; Hsieh et al., 2016, 2019; Shaltouki et al., 2018; Fang et al., 2019; Lautrup et al., 2019; Lou et al., 2020), and indicate that targeting mitophagy may have a broad application for disease intervention.

## Data Availability Statement

The original contributions presented in the study are included in the article/Supplementary Material, further inquiries can be directed to the corresponding author.

## Author Contributions

VB and C-HH designed and performed the experiments, analyzed the data, made the figures, and wrote the manuscript. XW conceived and supervised the project, designed the experiments, and wrote the manuscript. All authors contributed to the article and approved the submitted version.

## Conflict of Interest

XW is a co-founder, adviser, and shareholder of AcureX Therapeutics Inc., and a shareholder of Mitokinin Inc. C-HH is a shareholder of AcureX Therapeutics Inc. The remaining author declares that the research was conducted in the absence of any commercial or financial relationships that could be construed as a potential conflict of interest.

## Publisher’s Note

All claims expressed in this article are solely those of the authors and do not necessarily represent those of their affiliated organizations, or those of the publisher, the editors and the reviewers. Any product that may be evaluated in this article, or claim that may be made by its manufacturer, is not guaranteed or endorsed by the publisher.
