# Supplementary material for: Mitochondrial Defects in Fibroblasts of Pathogenic MAPT Patients
Source: Front Cell Dev Biol. 2021 Nov 3;9:765408. doi: 10.3389/fcell.2021.765408 (PMC8595217; doi:10.3389/fcell.2021.765408)

Mitochondrial Defects in Fibroblasts of Pathogenic *MAPT* Patients

Vinita Bharat [1], Chung-Han Hsieh [1], and Xinnan Wang [1]*

Affiliations:

1. Department of Neurosurgery, Stanford University School of Medicine, Stanford, CA94305, USA.

* Correspondence to: Xinnan Wang, Department of Neurosurgery, Stanford University School of Medicine, Palo Alto, CA 94305; xinnanw@stanford.edu

Supplementary Methods

ELISA

For Figure 1D, details are similar as in (Hsieh et al., 2019). Microplates (MaxiSorp, NUNC) were coated with mouse anti-Miro1 (clone 4H4, WH0055288M1, Sigma-Aldrich) at 1:1,000, or chicken anti-β-actin (LS-C82919, LifeSpan BioSciences) at 1:750 in 0.1 M sodium carbonate-bicarbonate buffer (3:7, pH=9.6) overnight at room temperature with cover to avoid evaporation. After plates were washed in wash buffer (0.05% Tween 20 in PBS, pH 7.3), nonspecific binding sites were blocked in PBS with 2% BSA (BP-1600-100, Fisher scientific) for 1 hr. Next, 50 µl of cell lysate prepared from above, or purified full-length Miro1 protein (0-900 ng/ml, ab163047, Abcam) was added and incubated at room temperature for 2 hrs. After washes, plates were incubated with biotinylated rabbit anti-Miro1 (ARP44818_P050, Aviva Systems Biology) at 1:1000, or biotinylated rabbit anti-β-actin (#5057S, Cell Signaling Technology) at 1:500, in 100 µl diluent (1% BSA in PBS, pH=7.3) for 2 hrs. Next, plates were washed and incubated with horseradish peroxidase-conjugated streptavidin (21130, Thermo Scientific) at 1:2000 in 100 µl diluent for 20 min. Plates were washed again, and 100 µl of the tetramethylbenzidine liquid substrate (SB01, Life

Technologies) was added and incubated for another 20 min. The colorimetric reactions were stopped by 50 µl 1 M H_2SO4 and absorbance was read at 450 nm by a microplate reader (FlexStation 3, Molecular devices). Miro1 value (ng/ml) was normalized to β-actin (OD450). The same assay was used for analyzing12 healthy subjects in (Hsieh et al., 2019). For Figure 1E-G, 4E, details are similar as in (Nguyen et al., 2021). The Rhot1 ELISA kit (EKL54911, Biomatik) was used according to the manufacturer's instructions. The specificity and stability were validated by Biomatik. The dynamic detection range, sensitivity (lower limit of detection), and precision (inter- and intra-assay) were determined by both Biomatik and us. Briefly, 50 µl of cell lysate prepared from above, or serial dilutions of the standard (0-40 ng/ml) were added and incubated for 2 hrs at 37°C. Each well was then incubated with 100 µl of Detection Reagent A for 1 hr at 37°C. Next, plates were washed, and each well was incubated with 100 µl of Detection Reagent B for 1 hr at 37°C. Plates were washed again, and 90 µl of Substrate Solution was added to each well for 15-25 min at 37°C. The colorimetric reactions were stopped by 50 µl of Stop Solution. Absorbance was read at 450 nm by a microplate reader (Infinite 200 Pro, Tecan). Miro1 value (ng/ml) was normalized to total protein concentration (100 µg/ml) measured by BCA. For all experiments, wells for generating the standard curve were included in each plate. Mann-Whitney *U* test was performed for comparing Miro1 signals within the same subject (DMSO v.s. CCCP).

Supplementary Figure Legends

Figure 1. Control Experiments. (A) Validation of our mitochondrial purification method. "Mito" and "Cyto" fractions from healthy fibroblasts (Healthy-6) were blotted as indicated. ATP5β is a mitochondrial marker. Calreticulin is an ER protein. (B) An IP similar to Figure 4C is shown. A negative control (empty vector) is included. All lanes are from the same blot.

Supplementary Figure 1

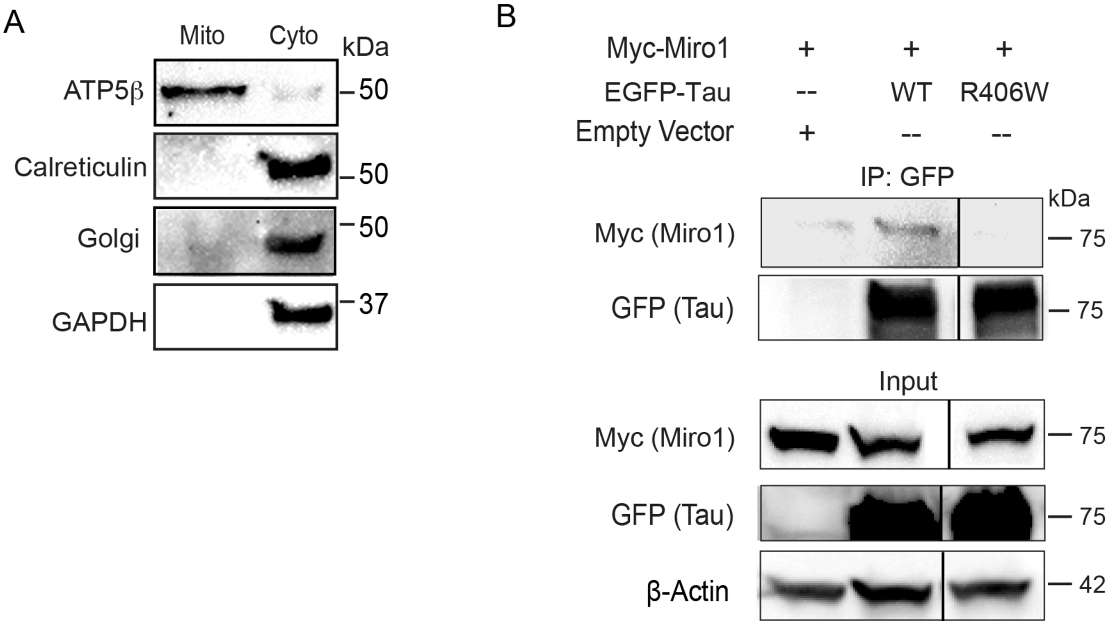

Supplement: Supplementary file 1 [file Presentation_1.PDF]
