# Supplementary material for: Mitochondrial Defects in Fibroblasts of Pathogenic MAPT Patients
Source: Front Cell Dev Biol. 2021 Nov 3;9:765408. doi: 10.3389/fcell.2021.765408 (PMC8595217; doi:10.3389/fcell.2021.765408)
Supplement: Supplementary file 2 [file Table_1.PDF]

ID	Name	Mutation	Age at Sampling (years)	Age at Onset (years)	Sex	Family History	UPDRS (sampling)	Hoehn and Yahr (sampling)	MMSE (sampling)	Source
1815	Healthy-1	wild-type	62		male					Dr. Pera
ND34769	Healthy-2	wild-type	68		female					NINDS
ND29510	Healthy-3	wild-type	55		female					NINDS
ND29178	Healthy-4	wild-type	66		male					NINDS
ND29179	Healthy-5	wild-type	68		male					NINDS
ND29194	Healthy-6	wild-type	51		female					NINDS
ND36091	Healthy-7	wild-type	63		female					NINDS
ND38530	Healthy-8	wild-type	55		male					NINDS
ND34770	Healthy-9	wild-type	72		male					NINDS
ND35044	Healthy-10	wild-type	77		male					NINDS
ND36320	Healthy-11	wild-type	71		female					NINDS
PPMI 3428	Healthy-12	wild-type	61		female		6			PPMI
ND40074	MAPT-1	MAPT (parkinsonism)	48	44	male	Y				NINDS
ND40070	MAPT-2	MAPT (parkinsonism)	43	43	female	Y	21	2	27	NINDS
ND40071	MAPT-3	MAPT (FTLD-P)	67	56	male	N			10	NINDS
ND40076	MAPT-4	MAPT (unaffected)	44		female	Y				NINDS
ND32955	MAPT-5	MAPT (unaffected)	50		male	Y				NINDS
ND32956	MAPT-6	MAPT (unaffected)	47		male	Y				NINDS
ND40072	MAPT-7	MAPT (unaffected)	36		female	Y			29	NINDS
